# Supplementary material for: Calcium sulfate beads made with antibacterial essential oil-water emulsions exhibit growth inhibition against Staphylococcus aureus in agar pour plates
Source: PLoS One. 2022 Jul 8;17(7):e0271209. doi: 10.1371/journal.pone.0271209 (PMC9269935; doi:10.1371/journal.pone.0271209)
Supplement: Attachment — Submitted filename: Response letter to the reviewers.pdf [file pone.0271209.s001.pdf]

Dear Reviewers,

Thank you for the opportunity to revise our manuscript **"Calcium sulfate beads made with antibacterial essential oil-water emulsions exhibit growth inhibition against *Staphylococcus aureus* in agar pour plates."** We appreciate your feedback and the feedback of the reviewers, we are also grateful for you and the reviewers taking the time to conduct a review process for this manuscript. We have considered the helpful comments and addressed them to the best of our ability. As requested in the decision letter email, we are providing responses to the items highlighted in the feedback about the manuscript.

Review Feedback and Comments pasted from decision letter email with our responses in red.

Please include the following items when submitting your revised manuscript:

- A rebuttal letter that responds to each point raised by the academic editor and reviewer(s). You should upload this letter as a separate file labeled 'Response to Reviewers'.
    - **Complete**
- A marked-up copy of your manuscript that highlights changes made to the original version. You should upload this as a separate file labeled 'Revised Manuscript with Track Changes'.
    - **Complete**
- An unmarked version of your revised paper without tracked changes. You should upload this as a separate file labeled 'Manuscript'.
    - **Complete**

If you would like to make changes to your financial disclosure, please include your updated statement in your cover letter. Guidelines for resubmitting your figure files are available below the reviewer comments at the end of this letter.

- N/A

If applicable, we recommend that you deposit your laboratory protocols in protocols.io to enhance the reproducibility of your results. Protocols.io assigns your protocol its own identifier (DOI) so that it can be cited independently in the future. For instructions see: http://journals.plos.org/plosone/s/submission-guidelines#loc-laboratory-protocols. Additionally, PLOS ONE offers an option for publishing peer-reviewed Lab Protocol articles, which describe protocols hosted on protocols.io. Read more information on sharing protocols at https://plos.org/protocols?utm_medium=editorial-email&utm_source=authorletters&utm_campaign=protocols.

- N/A

We look forward to receiving your revised manuscript.

Kind regards,

Arumugam Sundaramanickam, PhD
Academic Editor
PLOS ONE

Additional Editor Comments:

Most of the references are old. Include some recent references between 2019 and 2021.

- **Thank you. We updated all the references that could be updated. Some of the references, however, are seminal works or otherwise key pieces of information that**

support the narrative and project justification. If there are any specific references you would still like us to try to update, please let us know.

Journal Requirements:

Please review your reference list to ensure that it is complete and correct. If you have cited papers that have been retracted, please include the rationale for doing so in the manuscript text, or remove these references and replace them with relevant current references. Any changes to the reference list should be mentioned in the rebuttal letter that accompanies your revised manuscript. If you need to cite a retracted article, indicate the article's retracted status in the References list and also include a citation and full reference for the retraction notice.

When submitting your revision, we need you to address these additional requirements.

- We reformatted the references to match the formatting requirements of the journal.

1. Please ensure that your manuscript meets PLOS ONE's style requirements, including those for file naming. The PLOS ONE style templates can be found at

https://journals.plos.org/plosone/s/file?id=wjVg/PLOSOne_formatting_sample_main_body.pdf and

https://journals.plos.org/plosone/s/file?id=ba62/PLOSOne_formatting_sample_title_authors_affiliations.pdf

- **Thank you for this comment and the links. This was extremely helpful. We have revised the style of the manuscript exactly as instructed in the links. One issue we were unsure of, though, was table 1, which is quite wide. In order for table 1 to fit underneath the paragraph in which it is first mentioned, we had to flip the pages horizontally. We were not sure if this is appropriate or how we should approach this technical issue.**

2. Thank you for stating the following in the Acknowledgments Section of your manuscript:

"This work was supported by intramural funds from the FGCU Department of Biological Sciences and the FGCU Whitaker Center for Stem Education. We would like to thank doTERRA® International for the donation of essential oils in support of this work, especially Dr. David Hill, D.C. and Dr. Cody Beaumont, Ph.D. We would also like to thank Sean Aiken of Biocomposites ® (UK) for his gracious provision of bead making supplies and scientific input."

We note that you have provided funding information that is not currently declared in your Funding Statement. However, funding information should not appear in the Acknowledgments section or other areas of your manuscript. We will only publish funding information present in the Funding Statement section of the online submission form.

Please remove any funding-related text from the manuscript and let us know how you would like to update your Funding Statement. Currently, your Funding Statement reads as follows:

"The funders had no role in study design, data collection and analysis, decision to publish, or preparation of the manuscript."

Additionally, because some of your funding information pertains to commercial funding, we ask you to provide an updated Competing Interests statement, declaring all sources of commercial funding.

In your Competing Interests statement, please confirm that your commercial funding does not alter your adherence to PLOS ONE Editorial policies and criteria by including the following statement: "This does not alter our adherence to PLOS ONE policies on sharing data and materials." as detailed online in our guide for authors http://journals.plos.org/plosone/s/competing-interests. If this statement is not true and your adherence to PLOS policies on sharing data and materials is altered, please explain how.

Please include the updated Competing Interests Statement and Funding Statement in your cover letter. We will change the online submission form on your behalf.

Please know it is PLOS ONE policy for corresponding authors to declare, on behalf of all authors, all potential competing interests for the purposes of transparency. PLOS defines a competing interest as anything that interferes with, or could reasonably be perceived as interfering with, the full and objective presentation, peer review, editorial decision-making, or publication of research or non-research articles submitted to one of the journals. Competing interests can be financial or non-financial, professional, or personal. Competing interests can arise in relationship to an organization or another person. Please follow this link to our website for more details on competing interests: http://journals.plos.org/plosone/s/competing-interests

- Thank you. We have revised the acknowledgements section to read as follows:

"We would like to thank doTERRA® International for the donation of essential oils in support of this work, especially Dr. David Hill, D.C. and Dr. Cody Beaumont, Ph.D. We would also like to thank Sean Aiken of Biocomposites ® (UK) for his gracious provision of bead making supplies and scientific input."

3. We note that you have included the phrase "data not shown" in your manuscript. Unfortunately, this does not meet our data sharing requirements. PLOS does not permit references to inaccessible data. We require that authors provide all relevant data within the paper, Supporting Information files, or in an acceptable, public repository. Please add a citation to support this phrase or upload the data that corresponds with these findings to a stable repository (such as Figshare or Dryad) and provide and URLs, DOIs, or accession numbers that may be used to access these data. Or, if the data are not a core part of the research being presented in your study, we ask that you remove the phrase that refers to these data.

- Thank you. We have removed the 'data not shown" phrase from the manuscript. The manuscript is not significantly altered with/without it.

[Note: HTML markup is below. Please do not edit.]

Reviewers' comments:

Reviewer's Responses to Questions

Comments to the Author

1. Is the manuscript technically sound, and do the data support the conclusions?

The manuscript must describe a technically sound piece of scientific research with data that supports the

conclusions. Experiments must have been conducted rigorously, with appropriate controls, replication, and sample sizes. The conclusions must be drawn appropriately based on the data presented.

- **Thank you!**

Reviewer #1: Yes

Reviewer #2: Yes

---

2. Has the statistical analysis been performed appropriately and rigorously?

Reviewer #1: Yes

Reviewer #2: Yes

- **Thank you.**

---

3. Have the authors made all data underlying the findings in their manuscript fully available?

The PLOS Data policy requires authors to make all data underlying the findings described in their manuscript fully available without restriction, with rare exception (please refer to the Data Availability Statement in the manuscript PDF file). The data should be provided as part of the manuscript or its supporting information, or deposited to a public repository. For example, in addition to summary statistics, the data points behind means, medians and variance measures should be available. If there are restrictions on publicly sharing data—e.g. participant privacy or use of data from a third party—those must be specified.

Reviewer #1: Yes

Reviewer #2: Yes

- **Thank you.**

---

4. Is the manuscript presented in an intelligible fashion and written in standard English?

PLOS ONE does not copyedit accepted manuscripts, so the language in submitted articles must be clear, correct, and unambiguous. Any typographical or grammatical errors should be corrected at revision, so please note any specific errors here.

Reviewer #1: Yes

Reviewer #2: Yes

- **Thank you.**

---

5. Review Comments to the Author

Please use the space provided to explain your answers to the questions above. You may also include additional comments for the author, including concerns about dual publication, research ethics, or publication ethics. (Please upload your review as an attachment if it exceeds 20,000 characters)

Reviewer #1: This is a well done study with a clearly defined focus. It shows that anti-microbial oils may have potential for inhibiting S. aureus in calcium sufate beads. The results are also relevant, in addition to the medical aspects, in the food sector.

- **Thank you!**

Minor comment: line 77 and 79 S. aureus in italics.

- **Thanks. Corrected!**

Reviewer #2: The study is quite impressive and scientifically elaborated. I advise some minor corrections/clarifications:

- Mention the plants under study in the material and methods section. **DONE**

- Mention scientific names of the plants in Table 1 **DONE**

- Include some representative images of treatments displaying significantly higher ZOIs compared to vancomycin 30 µg control. **DONE. Added fig1**

-Include representative image of PEO seeded beads. **DONE Added fig2**

- What was the basis of selecting 30 µg of vancomycin as control? **30 µg is the minimum inhibitory concentration for S. aureus species and is used to conduct antibiograms in research and in the clinic to determine susceptibility/resistance. Also, the filter disks come pre-loaded with this amount of vancomycin, and a reference page for determining the susceptibility.**

- **Thank you for your compliment!**

---

6. PLOS authors have the option to publish the peer review history of their article (what does this mean?). If published, this will include your full peer review and any attached files.

If you choose “no”, your identity will remain anonymous but your review may still be made public.

Do you want your identity to be public for this peer review? For information about this choice, including consent withdrawal, please see our Privacy Policy.

Reviewer #1: **Yes:** Per Saris

- **Thank you!**

Reviewer #2: No

- **Thank you!**

[NOTE: If reviewer comments were submitted as an attachment file, they will be attached to this email and accessible via the submission site. Please log into your account, locate the manuscript record, and check for the action link "View Attachments". If this link does not appear, there are no attachment files.]

While revising your submission, please upload your figure files to the Preflight Analysis and Conversion Engine (PACE) digital diagnostic tool, https://pacev2.apexcovantage.com/. PACE helps ensure that figures meet PLOS requirements. To use PACE, you must first register as a user. Registration is free. Then, login and navigate to the UPLOAD tab, where you will find detailed instructions on how to use the tool. If you encounter any issues or have any questions when using PACE, please email PLOS at figures@plos.org. Please note that Supporting Information files do not need this step.

End of Responses

Once again, thank you for the review process and helpful comments and feedback. We look forward to publishing the manuscript. Please do not hesitate to reach out if we have missed anything, or if any additional feedback arises. We would be more than happy to address any other issues.

Most Sincerely,

Matthew C. Swearingen, PhD

p.s. Editor, this work was originally completed at Florida Gulf Coast University in Fort Myers, Florida. I have since left that institution and now work in private industry as a Senior Microbiologist. Hence, I am no longer using the FGCU contact information, but instead my personal information for correspondences.
