# Supplementary material for: Calcium sulfate beads made with antibacterial essential oil-water emulsions exhibit growth inhibition against Staphylococcus aureus in agar pour plates
Source: PLoS One. 2022 Jul 8;17(7):e0271209. doi: 10.1371/journal.pone.0271209 (PMC9269935; doi:10.1371/journal.pone.0271209)
Supplement: Attachment — Submitted filename: Response letter to the reviewers part 2.docx [file pone.0271209.s002.docx]

Dear (new) Editor and Reviewers,

Thank you for the opportunity to revise our manuscript **“Calcium sulfate beads made with antibacterial essential oil-water emulsions exhibit growth inhibition against *Staphylococcus aureus* in agar pour plates.”** We appreciate your feedback and the feedback of the reviewers, we are also grateful for you and the reviewers taking the time to conduct a review process for this manuscript. We have considered the helpful comments and addressed them to the best of our ability. As requested in the decision letter email, we are providing responses to the items highlighted in the feedback about the manuscript.

**Review Feedback and Comments pasted from decision letter email with our responses in red.**

Please include the following items when submitting your revised manuscript:

- Several monor error are presence (S. aureus not in italic line 74: "orthopedic pathogens, including S. aureus (10,14–16)."; ect...)
  - Fixed! Thank you!
- Is not reported the diameter of the plat used (90 mm or 120 mm).
  - Fixed line 105
- Introduction_Line 77-80: this are "result"...and cannot be insert in the introduction.
  - Fixed
- In the results there are repetition of "materials and methods"
  - Fixed lines 138-143 and 175-177

Please submit your revised manuscript by 31 March 2022. If you will need more time than this to complete your revisions, please reply to this message or contact the journal office at [plosone@plos.org](mailto:plosone@plos.org). When you're ready to submit your revision, log on to <https://www.editorialmanager.com/pone/> and select the 'Submissions Needing Revision' folder to locate your manuscript file.

Please include the following items when submitting your revised manuscript:

- A rebuttal letter that responds to each point raised by the academic editor and reviewer(s). You should upload this letter as a separate file labeled 'Response to Reviewers'.
  - complete
- A marked-up copy of your manuscript that highlights changes made to the original version. You should upload this as a separate file labeled 'Revised Manuscript with Track Changes'.
  - complete
- An unmarked version of your revised paper without tracked changes. You should upload this as a separate file labeled 'Manuscript'.
  - complete

If you would like to make changes to your financial disclosure, please include your updated statement in your cover letter. Guidelines for resubmitting your figure files are available below the reviewer comments at the end of this letter.

If applicable, we recommend that you deposit your laboratory protocols in [protocols.io](http://protocols.io/) to enhance the reproducibility of your results. Protocols.io assigns your protocol its own identifier (DOI) so that it can be cited independently in the future. For instructions see: <https://journals.plos.org/plosone/s/submission-guidelines#loc-laboratory-protocols>. Additionally, PLOS ONE offers an option for publishing peer-reviewed Lab Protocol articles, which describe protocols hosted on [protocols.io](http://protocols.io/). Read more information on sharing protocols at <https://plos.org/protocols?utm_medium=editorial-email&utm_source=authorletters&utm_campaign=protocols>.

We look forward to receiving your revised manuscript.

Kind regards,

Filippo Giarratana
Academic Editor
PLOS ONE

[Note: HTML markup is below. Please do not edit.]

Reviewers' comments:

Reviewer's Responses to Questions

**Comments to the Author**

1. If the authors have adequately addressed your comments raised in a previous round of review and you feel that this manuscript is now acceptable for publication, you may indicate that here to bypass the “Comments to the Author” section, enter your conflict of interest statement in the “Confidential to Editor” section, and submit your "Accept" recommendation.

Reviewer #1: All comments have been addressed

Reviewer #3: (No Response)

2. Is the manuscript technically sound, and do the data support the conclusions?

The manuscript must describe a technically sound piece of scientific research with data that supports the conclusions. Experiments must have been conducted rigorously, with appropriate controls, replication, and sample sizes. The conclusions must be drawn appropriately based on the data presented.

Reviewer #1: Yes

Reviewer #3: Partly

3. Has the statistical analysis been performed appropriately and rigorously?

Reviewer #1: Yes

Reviewer #3: No

4. Have the authors made all data underlying the findings in their manuscript fully available?

The [PLOS Data policy](http://www.plosone.org/static/policies.action#sharing) requires authors to make all data underlying the findings described in their manuscript fully available without restriction, with rare exception (please refer to the Data Availability Statement in the manuscript PDF file). The data should be provided as part of the manuscript or its supporting information, or deposited to a public repository. For example, in addition to summary statistics, the data points behind means, medians and variance measures should be available. If there are restrictions on publicly sharing data—e.g. participant privacy or use of data from a third party—those must be specified.

Reviewer #1: Yes

Reviewer #3: Yes

5. Is the manuscript presented in an intelligible fashion and written in standard English?

PLOS ONE does not copyedit accepted manuscripts, so the language in submitted articles must be clear, correct, and unambiguous. Any typographical or grammatical errors should be corrected at revision, so please note any specific errors here.

Reviewer #1: Yes

Reviewer #3: Yes

6. Review Comments to the Author

Please use the space provided to explain your answers to the questions above. You may also include additional comments for the author, including concerns about dual publication, research ethics, or publication ethics. (Please upload your review as an attachment if it exceeds 20,000 characters)

Reviewer #1: No further requirements.You have answered the requests in a proper manner. The work presents interesting results.

- Thank you

Reviewer #3: General comments:
Overall, I like the idea on which the work is based. The study tests the possible in vitro antimicrobial activity of several essential oils incorporated into calcium sulphate beads against S. aureus that is among the main causative agent of bone infections in orthopaedic medicine. the authors demonstrated a good command of scientific English. However, there are some limitations. Meanwhile, the results are described on the basis of a not well-defined statistical analysis. Or rather, in the tables this information is reported, however, there is no information about it in the materials and methods. After that, my main concern is that the authors tested multiple essential oils in the same plate. How can they be sure that there has not been an influence between the various oils? I understand that the halos of inhibition are well defined in the plates, however the diffusion of the essential oil in the medium certainly affected the growth.
These are important and relevant methodological limits for the reliability of the results.

Summary of Responses (followed by individual section responses)

- The student’s T test was used to determine statistical significance and this was previously written in the table 1 title in bold. The specific null hypotheses or otherwise were also described underneath the table.
  - I added a note about using student’s T test in each methods subsection lines 115 and 135.
  - Statistics were also previously written throughout the body of the manuscript, for example, lines 144 – 145
- Lines 188-192 address possible synergism/antagonism in multi-PEO plates. Although not within the scope of this study, **we** thank you for the call out and agree that acknowledging this limitation strengthens the discussion.

  Please check the punctuation and italic. I have the impression that a few dots are missing through the text (see lines 65 and 75,77,79 etc.).
- This was noted by the editor, and was completed. Thank you!

Introduction

This paragraph is well written and well explains the background on which the study moves. I appreciated how the aims achieved and the limits of experimentation are briefly mentioned in the final part.

- Thank you!

However, I suggest checking, and eventually rephrasing, the lines 79-81. Maybe, at the beginning of line 80, “or” was “of”? I understand what the Authors want to say but is a little bit confusing.

- Fixed. Thank you for the call-out.

Materials and methods

Line 101 “through preliminary testing”. What are you referring to? Where is this data? Explain better.

- Fixed the wording to not be less vague or misleading.

Line 102 “ZOIs”: is this the abbreviation of what? Do you mean zone of inhibition? It does not open to me is written by anyone else before. To specify. The same for other abbreviation used such as PPZOI in the results section.

- Defined ZOI line 111
- Defined PPZOI 133

Lines 101-102 “Some PEOs known to have high potency (through preliminary testing) were

- Fixed the wording, also addressed above

102 tested on plates in the absence” Were the other PEOs tested in common plates? If so, how can you be sure the effect of each PEO does not affect that of the other? Please, clarify.

- Lines 188-192 address possible synergism/antagonism in multi-PEO plates. Although not within the scope of this study, **we** thank you for the call out and agree that acknowledging this limitation strengthens the discussion.

I don't understand what kind of statistical analysis was done. Although there is some clarification in the tables, the type of statistical analysis that was performed is not specified in this section. Please, improve.

- I added a note about using student’s T test in each methods subsection lines 115 and 135.

Results

Overall, the data relating to the results are presented, however more than one sentence is not about the results but more about the materials and methods. In addition, there are also repetitions of the materials and methods. Results certainly need to be improved.

- Fixed

  Lines 135-139: These are not results; furthermore, these are repetitions of materials and methods. See also lines 189-191.
- Fixed

  Line 140: It is not clear how these statistically significant differences were calculated. What results were compared with each other? Are there any replicas of the samples? How much they were? In addition to reporting this information in the tables, it is necessary to report it in the text to understand not only the type of analysis used but whether it is actually possible to use this type of analysis for the type of data available. Specify and improve.
- Again, we used student’s t test and this is stated several times in the manuscript, and we now added lines to both methods subsections explicitly stating this. Table 1 was designed to comprehensively show the ZOI data and how comparisons used for p values.
  - For instance, a T test was used to determine that [for arborvitae] the disk diffusion assay ZOI was significantly higher than a vancomycin control.

    Discussion

    The discussions are well written, and the data reported in the results are properly argued. Furthermore, both the limitations and the innovations brought about by this study are highlighted.

    Figures

    The quality of the figures is poor. Improve.
  - This comment is subjective and/or too vague to enact specific change. The images were taken with a high resolution camera, and are at the maximum resolution of 300 dpi. Some oils do not elicit a perfect margin for ZOIs or especially PPZOIs which are 3 dimensional, and can create and impression of blurriness. At this point, I can’t retroactively improve image quality, nor is it acceptable to “doctor” images.

7. PLOS authors have the option to publish the peer review history of their article ([what does this mean?](https://journals.plos.org/plosone/s/editorial-and-peer-review-process#loc-peer-review-history)). If published, this will include your full peer review and any attached files.


If you choose “no”, your identity will remain anonymous but your review may still be made public.


**Do you want your identity to be public for this peer review?** For information about this choice, including consent withdrawal, please see our [Privacy Policy](https://www.plos.org/privacy-policy).

Reviewer #1: No

Reviewer #3: No

Once again, thank you for the review process and helpful comments and feedback. We look forward to publishing the manuscript. Please do not hesitate to reach out if we have missed anything, or if any additional feedback arises. We would be more than happy to address any other issues.

Most Sincerely,

Matthew C. Swearingen, PhD

p.s. Editor, this work was originally completed at Florida Gulf Coast University in Fort Myers, Florida. I have since left that institution and now work in private industry as a Senior Microbiologist. Hence, I am no longer using the FGCU contact information, but instead my personal information for correspondences.
